# Prevalence and Antibiotic Susceptibility of Bacterial Isolates From Dogs With Ulcerative Keratitis in Midwestern United States

**DOI:** 10.3389/fvets.2020.583965

**Published:** 2020-11-20

**Authors:** Joshua S. Hewitt, Rachel A. Allbaugh, Danielle E. Kenne, Lionel Sebbag

**Affiliations:** ^1^Department of Veterinary Clinical Sciences, College of Veterinary Medicine, Iowa State University, Ames, IA, United States; ^2^Veterinary Diagnostic Laboratory, College of Veterinary Medicine, Iowa State University, Ames, IA, United States

**Keywords:** bacterial keratitis, canine, topical antibiotic, systemic antibiotic, antibiotic resistance, multidrug resistance

## Abstract

The objective of this study was to describe bacterial culture and antibiotic susceptibility results in 476 dogs presenting with suspected bacterial keratitis in Iowa and surrounding Midwestern states, further detailing trends in patient characteristics, seasonality, and antimicrobial resistance. Corneal swabs yielded 465 bacterial isolates and 220 cultures (46.2%) with no apparent growth (0–5 isolates per culture). The most frequent bacterial genera were *Staphylococcus* (32.3%), *Streptococcus* (19.1%), and *Pseudomonas* (12.5%), while the most common bacterial species were *Staphylococcus pseudintermedius* (26.7%), *Streptococcus canis* (12%), and *Pseudomonas aeruginosa* (7.5%). Compared to mixed-breed dogs, canine breeds most likely to be examined for ulcerative keratitis included Boston terrier, Cavalier King Charles spaniel, miniature pinscher, pug, rat terrier, Saint Bernard, shih tzu, and silky terriers. In summer, the likelihood to yield a negative culture was reduced while the likelihood to culture *Pseudomonas* species was increased. Bacteria considered multidrug resistant (MDR, resistant to ≥ 3 antibiotic classes) represented 20% of all canine isolates and were most prevalent for *Staphylococcus* species (33%). An alarming, escalating trend of MDR prevalence was noted between 2016 (5%) and 2020 (34%). Individual ophthalmic preparations (i.e., single antibiotics or commercially available antibiotic combinations) with highest efficacy against all bacterial isolates included chloramphenicol (83%), ceftiofur (79%), amikacin (77%), neomycin-polymyxin B-bacitracin (77%), and gentamicin (74%). Efficacy of systemic antibiotics and combinations of ophthalmic preparations was also evaluated. Based on the present findings, triple antibiotic (Neo-Poly-Bac) is recommended as empirical monotherapy for prophylactic antibiotic therapy in dogs with simple corneal ulcers, while a chloramphenicol-ciprofloxacin combination is empirically recommended for therapeutic management of infected corneal ulcers. Pending culture and susceptibility results, appropriate selection of empiric antibiotic therapy is important to enhance therapeutic outcome and reduce antibacterial resistance in dogs with corneal ulceration.

## Introduction

Bacterial keratitis is a major global cause of ocular discomfort and visual impairment in dogs and other species. Following an injury to the eye from trauma or other causes, corneal wounds in dogs have a high tendency toward infection given the presence of indigenous microflora on the corneal and conjunctival surfaces, including *Staphylococcus* species, *Streptococcus* species, *Pseudomonas* species, and gram-positive bacilli (1–3). Rapid and appropriate use of antibiotics is critical in mitigating the potential devastating effects of bacterial keratitis in dogs, including keratomalacia (corneal melting), corneal perforation, scarring, and loss of vision or the entire eye (4, 5). Successful management requires the clinician to be aware of the most common bacterial isolates and their susceptibility profiles to antibiotics, as there is often a lag period of several days until corneal samples collected from the patient provide results for bacterial culture and sensitivity testing.

The distribution of bacterial populations has been described previously for several geographic regions of the world including Asia (3, 6, 7), Australia (1, 8), Europe (9), North America (10, 11), and South America (2). While there are distinct similarities between these regional investigations, there are notable differences in bacterial prevalence and susceptibilities across the world. Further, differences within separate geographic regions of even a single country (10, 11) demonstrate that more targeted evaluation of local or regional populations is needed. Most previous reports in veterinary literature are limited to describing the prevalence of bacterial isolates and susceptibility profiles for individual antibiotics (6, 7, 10). Such information is useful but is generally insufficient to provide optimized recommendations for clinicians managing bacterial keratitis in dogs. For instance, it is common practice to use two different ophthalmic antibiotics in veterinary patients with an infected corneal ulcer (12–15) or reach for systemic antibiotics in patients with vascularized corneal lesions or corneal perforations. However, to our knowledge, specific comparisons of antibiotic combination efficacies on bacterial isolates has not been described in dogs or other species, and only selected canine reports have described the efficacy of systemic antibiotics for corneal disease (16, 17). Using fewer, more effective drugs for an appropriate timeframe allows for better patient outcomes and less antibiotic resistance. Less antibiotic resistance in the environment will benefit both veterinary and human medicine.

The primary goal of the study was to describe results of bacterial cultures and susceptibility testing (ophthalmic and systemic profiles) in canine patients that presented with bacterial keratitis from Iowa and surrounding Midwestern states of the United States. A secondary objective was to report the prevalence and trends over time (2014–2020) of antibacterial resistance in corneal isolates, as well as risk factors associated with positive bacterial growth from corneal samples. We hope this information will provide clinically relevant data for managing bacterial keratitis in dogs and help mitigate the alarming rise of antibacterial resistance in the species.

## Materials and Methods

### Data Collection

The database of Iowa State University's Veterinary Diagnostic Laboratory (ISU VDL) was searched for results of bacterial cultures collected from canine corneas, as well as associated antibiotic susceptibility testing when available. The search covered a period from March 2014—when an ophthalmic-specific susceptibility profile was introduced—to the date of manuscript writing (June 2020). Corneal cultures processed by ISU VDL originated from two sources: (i) In-house submissions from the ISU Lloyd Veterinary Medical Center (ISU LVMC), for which information about patients characteristics (i.e., age, breed) was available; and (ii) Mail-in submissions from veterinary practices in Iowa and surrounding states (Minnesota, Missouri, South Dakota).

### Sample Identification and Susceptibility Testing

At the ISU LVMC, samples were collected with sterile culturette swabs (BBL^TM^ CultureSwab^TM^, Becton Dickinson and Company, Sparks, MD) that were pre-moistened prior to contact with the corneal wound and processed for aerobic microbiologic assessment using a non-selective medium (tryptic soy agar with 5% sheep blood [blood agar]) and a Gram-negative selective medium (MacConkey). The blood agar was incubated at 35 ± 2°C with 5–10% CO_2_ for a total length of 4 days while the MacConkey agar was incubated 35 ± 2°C without CO_2_ for a total length of 2 days. Both agar plates were observed for growth every 24 h. Organisms were then identified using Matrix-Assisted Laser Desorption Ionization Time-of-Flight mass-spectrometry (MALDI-TOF MS, Bruker) or conventional microbiology methods when necessary. Minimum inhibitory concentration (MIC) susceptibility testing was performed using an automated broth microdilution system (Sensititre AIM, Trek Diagnostic System Inc.) and susceptibility panels (Thermo Fisher Scientific). Interpretations were determined by the MIC breakpoints, which are based on the VET08 and M100 Clinical and Laboratory Standards Institute (CLSI) documents (18, 19). Depending on the clinician's request, susceptibility testing performed by the ISU VDL included an ophthalmic susceptibility profile (JOEYE2 plate, Thermo Scientific Inc.) and/or a systemic susceptibility profile. Antibiotics and drug concentrations evaluated in JOEYE2 plates can be found on the manufacturer's website (assets.thermofisher.com/TFS-Assets/MBD/Specification-Sheets/Sensititre-Plate-Layout-JOEYE2.pdf).

### Data Analysis

Results considered “non-interpretable” by CLSI guidelines were excluded from data analysis, that is, not classified as susceptible nor resistant in calculations of percent sensitivity and multidrug resistance. An isolate was considered susceptible to a combination therapy (e.g., chloramphenicol-ciprofloxacin) if one or both antibiotics yielded a “susceptible” result for the given isolate. Bacterial isolates were considered multidrug resistant (MDR) if resistant to three or more classes of antibiotics (20), removing all known intrinsic resistances from the MDR definition as described by Sweeney et al. (21).

Odds ratios were calculated with SigmaPlot 14.0 (Systat software, Point Richmond, CA), and *P* < 0.05 were considered significant unless another α-value is described: (i) Likelihood of a pure breed *vs*. mixed breed dog to present to the ISU LVMC Ophthalmology service with a corneal infection—as compared to other reasons for the visit such as keratoconjunctivitis sicca, glaucoma, or cataracts—evaluating the canine population presented to Ophthalmology over the 6-years study period; (ii) Likelihood of a pure breed *vs*. mixed breed dog with a corneal infection (i.e., positive corneal culture) to yield a bacterial isolate classified as MDR; (iii) Likelihood of selected antibiotics vs. all others to provide higher efficacy against all bacterial isolates, adjusting the α-value to 0.0026 (0.05/19) to account for multiple pairwise comparisons with the Bonferroni correction; and (iii) Likelihood of not detecting bacteria (i.e., negative corneal culture) or detecting selected bacterial genera in different seasons. For the latter, a corneal culture performed between March and May was considered as the spring season, June to August for summer, September to November for fall, and December to February for winter.

## Results

### Patients Characteristics

Between March 2014 and June 2020, corneal swabs were obtained from 476 dogs with suspected bacterial keratitis and submitted in-house (ISU LVMC, *n* = 317) or as mail-in (referring clinics, *n* = 159) to ISU VDL for aerobic bacterial culture and susceptibility testing. The population was comprised of dogs age 2 months to 17 years old, including 57 (8%) intact male, 285 (41%) castrated male, 56 (8%) intact female, 263 (38%) spayed female dogs, and 30 (4%) patients with no sex listed. Mixed breed dogs represented 17% (79/476) of the study population, while the most common pure breeds were shih tzu (18%, 86/476), Boston terriers (9%, 41/476), and Yorkshire terriers (4%, 18/476). Compared to mixed breed dogs, Labrador retrievers had a significantly lower likelihood (OR = 0.10, 95% CI = 0.03–0.28, *P* < 0.001) to present to the ISU LVMC Ophthalmology service with a suspected bacterial infection, while the likelihood was significantly higher in the following canine breeds: Boston terriers (OR = 4.16, 95% CI = 2.14–8.09, *P* < 0.001), Cavalier King Charles spaniels (OR = 4.55, 95% CI = 1.63–12.65, *P* = 0.006), miniature pinschers (OR = 6.77, 95% CI = 2.56–17.90, *P* < 0.001), pugs (OR = 3.93, 95% CI = 1.75–8.80, *P* = 0.001), rat terriers (OR = 5.31, 95% CI = 2.04–13.83, *P* < 0.001), Saint Bernards (OR = 16.37, 95% CI = 2.85–93.95, *P* = 0.002), shih tzus (OR = 6.61, 95% CI = 3.92–11.13, *P* < 0.001), and silky terriers (OR = 10.91, 95% CI = 2.09–57.01, *P* = 0.012).

### Bacterial Isolate Characteristics

**Figure 1:**
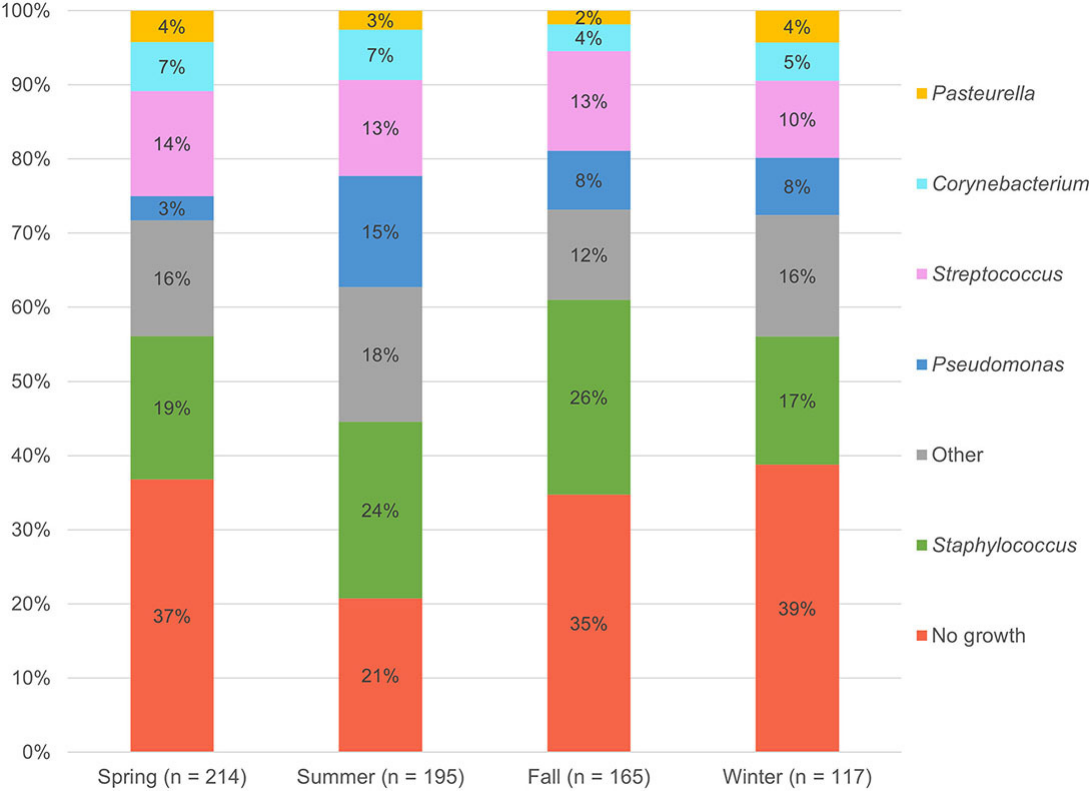
Percentage of bacterial genera isolated per season.

The 476 aerobic cultures resulted in 465 bacterial isolates, six fungal growths (data not shown), and 220 cultures (46.2%) with no apparent growth (0–5 isolates per culture). The proportion of negative bacterial cultures did not differ between in-house samples (147/317, 46.4%) and mail-in samples (73/159, 45.9%). Table 1 provides a summary of the bacterial genera and species isolated from canine corneas. The most common bacterial genera were *Staphylococcus* species (150/465, 32.3%), *Streptococcus* species (89/465, 19.1%), *Pseudomonas* species (58/465, 12.5%), and *Corynebacterium* species (39/465, 8.4%), while the most common bacterial species included *Staphylococcus pseudintermedius* (124/465, 26.7%), *Streptococcus canis* (56/465, 12.0%), and *Pseudomonas aeruginosa* (35/465, 7.5%). Further information detailing the species of bacteria isolated from canine corneas is summarized in Table 1. The number of isolates cultured in the spring (*n* = 134), summer (*n* = 153), fall (*n* = 107), and winter (*n* = 71) are described by genera in Figure 1. Compared to all bacterial isolates, *Pseudomonas* species were significantly less likely to be isolated in the spring (OR = 0.34, 95% CI = 0.15–0.77, *P* = 0.011), but were significantly more likely to be isolated in the summer (OR = 2.04, 95% CI = 1.18–3.53, *P* = 0.015). Compared to all bacterial cultures, a negative culture result (no growth) was significantly less likely to occur in the summer (OR = 0.57, 95% CI = 0.39–0.83, P = 0.004).

**Table 1:** Bacterial species isolated from suspected clinically infected canine corneas.

**Organism**	**# of isolates**	**Proportion (%)**
*Staphylococcus* species	(150)	(32.3)
*Staphylococcus pseudintermedius*	124	26.7
Coagulase(-) *Staphylococcus* (non-specified)	14	3.0
*Staphylococcus epidermidis*	3	0.6
*Staphylococcus aureus*	2	0.4
*Staphylococcus schleiferi*	2	0.4
*Staphylococcus* sp. (non-specified)	2	0.4
Coagulase(+) *Staphylococcus* (non-specified)	1	0.2
*Staphylococcus delphinus*	1	0.2
*Staphylococcus sciuri*	1	0.2
*Streptococcus* species	(89)	(19.1)
*Streptococcus canis*	56	12.0
Alpha-hemolytic S*treptococcus* (non-specified)	14	3.0
*Streptococcus* sp. (non-specified)	9	1.9
*Streptococcus agalactiae*	3	0.6
*Streptococcus dysgalactiae* ss. *equisimilis*	2	0.4
*Streptococcus oralis*	2	0.4
Beta-hemolytic *Streptococcus* (non-specified)	1	0.2
*Streptococcus salivarius*	1	0.2
*Streptococcus sanguis*	1	0.2
*Pseudomonas* species	(58)	(12.5)
*Pseudomonas aeruginosa*	35	7.5
*Pseudomonas* sp. (non-specified)	23	4.9
*Corynebacterium* species	39	8.4
*Pasteurella* species	22	4.7
*Escherichia coli*	13	2.8
*Actinomyces* species	10	2.2
*Bacillus* species	10	2.2
Other^a^	74	15.9
Total	465	100
No growth	220	

a *Other organisms include Enterococcus sp. (9), gram(-) non-fermenter (non-specified) (9), gram(-) rod (non-specified) (7), gram(+) rod (non-specified) (5), Acinetobacter sp. (4), Moraxella sp. (4), Neisseria sp. (4), Enterobacter sp. (3), gram(+) coccus (non-specified) (3), Micrococcus sp. (3), Proteus mirabilis (3), Serratia marcescens (3), Cutibacterium acnes (2), Klebsiella sp. (2), Achromobacter xylosoxidans (1), Clostridium perfringens (1), Capnocytophaga sp. (1), Cardiobacterium sp. (1), Chryseobacterium sp. (1), gram(-) coccus (non-specified) (1), Mycoplasma sp. (1), Pantoea agglomerans (1), Providencia rettgeri (1), Psychrobacter sp. (1), Raoultella sp. (1), Rhodococcus sp. (1), Rothia sp. (1)*.

### Topical and Systemic Antibiotic Susceptibility Testing by MIC

Ophthalmic and systemic susceptibility profiles were available for 352 and 308 of the isolates, respectively. Table 2 summarizes the findings from the ophthalmic susceptibility profiles, describing results of individual antibiotics as well as multi-drug formulations commonly used in veterinary practice (neomycin-polymyxin B-bacitracin, oxytetracycline-polymyxin B, and polymyxin B-trimethoprim). Except for erythromycin (30%), all antibiotics tested had a significantly (*P* ≤ 0.0026) greater percentage of susceptible isolates compared to polymyxin B (0%), bacitracin (7%), cefazolin (8%), and moxifloxacin (29%). Further, bacterial isolates were significantly (*P* < 0.0026) more susceptible to gentamicin (74%), neomycin (76%), amikacin (77%), ceftiofur (79%), and chloramphenicol (83%) compared to all other ophthalmic antibiotics tested except ciprofloxacin (69%). Minimum inhibitory concentrations required to inhibit the growth of 50 or 90% of organisms (MIC_50_ and MIC_90_, respectively) are described in Table 3. For the systemic susceptibility profile, the highest efficacy was seen with vancomycin (90%), imipenem (86%), and amikacin (80%), while the lowest efficacy was seen with pradofloxacin (0%), cephalexin (23%), and orbifloxacin (30%) (Table 4).

**Table 2:** *In vitro* susceptibility of 352 bacterial isolates to potential topical ophthalmic antibiotics.

**Antibiotic**	***Corynebacterium***	***Escherichia***	**Other^**a**^**	***Pasteurella* sp**.	***Pseudomonas* sp**.	***Staphylococcus* sp**.	***Streptococcus* sp**.	**Total**
	**sp**.	***coli***						
Amikacin	96% (26/27)	90% (9/10)	78% (49/63)	88% (15/17)	93% (41/44)	98% (120/123)	16% (11/68)	77% (271/352)
Bacitracin	48% (13/27)	0% (0/10)	11% (7/63)	0% (0/17)	2% (1/44)	0% (0/123)	3% (2/68)	7% (23/352)
Cefazolin	11% (3/27)	0% (0/10)	13% (8/63)	12% (2/17)	0% (0/44)	7% (9/123)	9% (6/68)	8% (28/352)
Ceftiofur	81% (22/27)	100% (10/10)	75% (47/63)	94% (16/17)	20% (9/44)	88% (108/123)	99% (67/68)	79% (279/352)
Chloramphenicol	93% (25/27)	100% (10/10)	87% (55/63)	94% (16/17)	20% (9/44)	88% (108/123)	100% (68/68)	83% (291/352)
Ciprofloxacin	78% (21/27)	100% (10/10)	56% (35/63)	29% (5/17)	95% (42/44)	79% (97/123)	47% (32/68)	69% (242/352)
Doxycycline	96% (26/27)	90% (9/10)	48% (30/63)	94% (16/17)	27% (12/44)	54% (66/123)	35% (24/68)	52% (183/352)
Erythromycin	67% (18/27)	0% (0/10)	13% (8/63)	47% (8/17)	0% (0/44)	59% (72/123)	1% (1/68)	30% (107/352)
Gentamicin	89% (24/27)	100% (10/10)	83% (52/63)	94% (16/17)	98% (43/44)	72% (89/123)	41% (28/68)	74% (262/352)
Moxifloxacin	4% (1/27)	0% (0/10)	3% (2/63)	0% (0/17)	0% (0/44)	80% (99/123)	1% (1/68)	29% (103/352)
Neomycin	100% (27/27)	90% (9/10)	83% (52/63)	100% (17/17)	93% (41/44)	89% (109/123)	19% (13/68)	76% (268/352)
Neomycin/Polymyxin B/Bacitracin	100% (27/27)	90% (9/10)	83% (52/63)	100% (17/17)	93% (41/44)	89% (109/123)	22% (15/68)	77% (270/352)
Ofloxacin	41% (11/27)	50% (5/10)	27% (17/63)	29% (5/17)	45% (20/44)	80% (98/123)	46% (31/68)	53% (187/352)
Oxytetracycline	85% (23/27)	80% (8/10)	78% (49/63)	100% (17/17)	41% (18/44)	54% (66/123)	63% (43/68)	64% (224/352)
Oxytetracycline/Polymyxin B	85% (23/27)	80% (8/10)	78% (49/63)	100% (17/17)	41% (18/44)	54% (66/123)	63% (43/68)	64% (224/352)
Polymyxin B	0% (0/27)	0% (0/10)	0% (0/63)	0% (0/17)	0% (0/44)	0% (0/123)	0% (0/68)	0% (0/352)
Polymyxin B/ Trimethoprim	89% (24/27)	90% (9/10)	44% (28/63)	12% (2/17)	18% (8/44)	74% (91/123)	18% (12/68)	49% (174/352)
Ticarcillin	44% (12/27)	80% (8/10)	54% (34/63)	29% (5/17)	64% (28/44)	40% (49/123)	56% (38/68)	49% (174/352)
Tobramycin	44% (12/27)	100% (10/10)	51% (32/63)	29% (5/17)	100% (44/44)	76% (93/123)	6% (4/68)	57% (200/352)
Trimethoprim/Sulfamethoxazole	89% (24/27)	90% (9/10)	44% (28/63)	12% (2/17)	18% (8/44)	74% (91/123)	18% (12/68)	49% (174/352)

a *Other organisms include Actinomyces sp. (8), Enterococcus sp. (6), Acinetobacter sp. (4), Enterobacter sp. (4), gram(+) organism (non-specified) (4), gram(-) non-fermenter (non-specified) (4), Moraxella sp. (4), Neisseria sp. (4), gram(-) organism (non-specified) (3), Micrococcus sp. (3), Proteus mirabilis (3), Serratia marcescens (3), Bacillus sp. (2), Cutibacterium acnes (2), Klebsiella sp. (2), Achromobacter xylosoxidans (1), Capnocytophaga sp. (1), Cardiobacterium sp. (1), Chryseobacterium sp. (1), Providencia rettgeri (1), Rhodococcus sp. (1), Rothia sp. (1)*.

**Table 3:** Minimal inhibitory concentrations (MIC_50_ and MIC_90_) of ophthalmic antibiotics for the most prevalent bacterial genera isolated in dogs with bacterial keratitis.

	***Staphylococcus*** **sp. (*****n*** **=** **123)**				***Streptococcus*** **sp. (*****n*** **=** **68)**				***Pseudomonas*** **sp. (*****n*** **=** **44)**			
	**MIC_**50**_**	**MIC_**90**_**	**Susceptible**	**Resistant**	**MIC_**50**_**	**MIC_**90**_**	**Susceptible**	**Resistant**	**MIC_**50**_**	**MIC_**90**_**	**Susceptible**	**Resistant**
Amikacin	≤16	≤16	98%	0%	>32	>32	16%	53%	≤16	≤16	93.2%	0.0%
Bacitracin	>4	>4	0%	41%	>4	>4	0%	0%	>4	>4	0.0%	0.0%
Cefazolin	≤8	≤8	7%	10%	≤8	≤8	9%	3%	>16	>16	0.0%	81.8%
Ceftiofur	≤2	≤2	88%	7%	≤2	≤2	99%	1%	>4	>4	20.5%	79.5%
Chloramphenicol	≤4	>16	88%	12%	≤4	≤4	100%	0%	>16	>16	20.5%	77.3%
Ciprofloxacin	≤1	>4	79%	20%	≤1	2	47%	3%	≤1	≤1	95.5%	2.3%
Doxycycline	0.25	>2	54%	0%	0.25	>2	35%	0%	>2	>2	27.3%	0.0%
Erythromycin	≤0.5	>4	59%	39%	≤0.5	2	1%	15%	>4	>4	0.0%	4.5%
Gentamicin	≤2	>8	72%	20%	8	>8	41%	13%	≤2	≤2	97.7%	0.0%
Moxifloxacin	≤0.5	>1	80%	17%	≤0.5	≤0.5	1%	0%	1	1	0.0%	0.0%
Neomycin	≤4	>8	89%	11%	>8	>8	19%	79%	≤4	8	93.2%	4.5%
Ofloxacin	0.5	>1	80%	14%	1	>1	46%	0%	0.5	1	45.5%	0.0%
Oxytetracycline	1	>4	54%	22%	2	>4	63%	22%	>4	>4	40.9%	38.6%
Polymyxin B	10	>10	0%	36%	>10	>10	0%	56%	≤5	≤5	0.0%	0.0%
Ticarcillin	≤16	≤16	40%	7%	≤16	≤16	56%	0%	≤16	64	63.6%	6.8%
Tobramycin	≤4	16	76%	14%	8	16	6%	24%	≤4	≤4	100.0%	0.0%
Trimethoprim-Sulfamethoxazole	≤2	>2	74%	26%	≤2	≤2	18%	0%	>2	>2	18.2%	20.5%

**Table 4:** *In vitro* susceptibility of 308 bacterial isolates to systemically distributed antibiotics.

**Antibiotic**	***Corynebacterium* sp**.	***Escherichia coli***	**Other^**a**^**	***Pasteurella* sp**.	***Pseudomonas* sp**.	***Staphylococcus* sp**.	***Streptococcus* sp**.	**Total**
Amikacin	100% (28/28)	100% (10/10)	78% (40/51)	100% (18/18)	100% (34/34)	99% (108/109)	16% (9/58)	80% (247/308)
Amoxicillin/Clavulanic acid	82% (23/28)	60% (6/10)	94% (48/51)	100% (18/18)	18% (6/34)	73% (80/109)	100% (58/58)	78% (239/308)
Ampicillin	32% (9/28)	50% (5/10)	57% (29/51)	83% (15/18)	0% (0/34)	54% (59/109)	83% (48/58)	54% (165/308)
Cefazolin	71% (20/28)	70% (7/10)	61% (31/51)	67% (12/18)	9% (3/34)	73% (80/109)	93% (54/58)	67% (207/308)
Cefovecin	46% (13/28)	60% (6/10)	43% (22/51)	28% (5/18)	0% (0/34)	38% (41/109)	60% (35/58)	40% (122/308)
Cefoxitin	23% (3/13)	100% (5/5)	78% (14/18)	43% (3/7)	0% (0/12)	77% (41/53)	39% (9/23)	57% (75/131)
Cefpodoxime	39% (11/28)	100% (10/10)	45% (23/51)	28% (5/18)	0% (0/34)	42% (46/109)	84% (49/58)	47% (144/308)
Ceftazidime		100% (5/5)	72% (13/18)	18% (2/11)	100% (22/22)			75% (42/56)
Ceftiofur	62% (8/13)	100% (5/5)	89% (16/18)	100% (7/7)	25% (3/12)	77% (41/53)	96% (22/23)	78% (102/131)
Cephalothin	86% (24/28)		64% (14/22)			74% (80/108)	92% (48/52)	79% (166/210)
Cephalexin		100% (5/5)	33% (6/18)	18% (2/11)	0% (0/22)			23% (13/56)
Chloramphenicol	89% (25/28)	100% (10/10)	84% (43/51)	94% (17/18)	18% (6/34)	82% (89/109)	38% (22/58)	69% (212/308)
Clindamycin	68% (19/28)	0% (0/5)	42% (14/33)	14% (1/7)	8% (1/12)	64% (70/109)	84% (49/58)	61% (154/252)
Doxycycline	89% (25/28)	90% (9/10)	61% (31/51)	67% (12/18)	38% (13/34)	56% (61/109)	38% (22/58)	56% (173/308)
Enrofloxacin	57% (16/28)	100% (10/10)	63% (32/51)	94% (17/18)	59% (20/34)	76% (83/109)	31% (18/58)	64% (196/308)
Erythromycin	71% (20/28)	0% (0/5)	27% (9/33)	29% (2/7)	0% (0/12)	61% (66/109)	48% (28/58)	50% (125/252)
Gentamicin	89% (25/28)	100% (10/10)	78% (40/51)	100% (18/18)	100% (34/34)	68% (74/109)	43% (25/58)	73% (226/308)
Imipenem	82% (23/28)	100% (10/10)	94% (48/51)	94% (17/18)	85% (29/34)	74% (81/109)	98% (57/58)	86% (265/308)
Marbofloxacin	64% (18/28)	100% (10/10)	69% (35/51)	94% (17/18)	94% (32/34)	82% (89/109)	52% (30/58)	75% (231/308)
Minocycline	53% (8/15)		60% (9/15)			57% (32/56)	49% (17/35)	55% (66/121)
Nitrofurantoin	0% (0/15)		33% (5/15)			96% (54/56)	71% (25/35)	69% (84/121)
Orbifloxacin		100% (5/5)	39% (7/18)	18% (2/11)	14% (3/22)			30% (17/56)
Oxacillin	21% (6/28)	0% (0/5)	30% (10/33)	29% (2/7)	0% (0/12)	72% (79/109)	57% (33/58)	52% (130/252)
Penicillin	14% (4/28)	0% (0/5)	33% (11/33)	29% (2/7)	0% (0/12)	28% (31/109)	71% (41/58)	35% (89/252)
Piperacillin		100% (5/5)	72% (13/18)	18% (2/11)	100% (22/22)			75% (42/56)
Pradofloxacin	0% (0/15)	0% (0/5)	0% (0/33)	0% (0/11)	0% (0/22)	0% (0/56)	0% (0/35)	0% (0/177)
Rifampin	71% (20/28)	0% (0/5)	12% (4/33)	0% (0/7)	0% (0/12)	72% (79/109)	0% (0/58)	41% (103/252)
Tetracycline	73% (11/15)	80% (4/5)	27% (9/33)	0% (0/11)	0% (0/22)	46% (26/56)	14% (5/35)	31% (55/177)
Ticarcillin	31% (4/13)	80% (4/5)	67% (12/18)	43% (3/7)	67% (8/12)	42% (22/53)	43% (10/23)	48% (63/131)
Ticarcillin/Clavulanic acid	31% (4/13)	80% (4/5)	78% (14/18)	43% (3/7)	75% (9/12)	42% (22/53)	43% (10/23)	50% (66/131)
Trimethoprim-Sulfamethoxazole	82% (23/28)	100% (10/10)	45% (23/51)	83% (15/18)	15% (5/34)	71% (77/109)	16% (9/58)	53% (162/308)
Vancomycin	100% (15/15)		27% (4/15)			98% (55/56)	100% (35/35)	90% (109/121)

a *Other organisms include Actinomyces sp. (7), Enterococcus sp. (7), Acinetobacter sp. (5), Enterobacter sp. (4), gram(-) organism (non-specified) (4), Moraxella sp. (4), Neisseria sp. (3), Proteus mirabilis (3), Cutibacterium acnes (2), gram(+) organism (non-specified) (2), Micrococcus sp. (2), Serratia marcescens (2), Achromobacter xylosoxidans (1), Bacillus sp. (1), Capnocytophaga sp. (1), Cardiobacterium sp. (1), Raoultella sp. (1), Rothia sp. (1)*.

### Combination Therapies

Table 5 describes the efficacy of combining two antibiotic formulations (reported in the ophthalmic susceptibility) against all bacterial isolates identified in the study for which an ophthalmic panel was available (*n* = 352), considering individual antibiotics as well as commercially available combination formulations. The most effective combinations were amikacin-chloramphenicol (98% susceptible), chloramphenicol-neomycin (98%), and amikacin-ceftiofur (97%), while the least effective combinations were bacitracin-polymyxin B (7% susceptible), cefazolin-polymyxin B (8%), and bacitracin-cefazolin (13%).

**Table 5:** Susceptibility of 352 canine corneal isolates to one or both indicated antibiotics^a^.

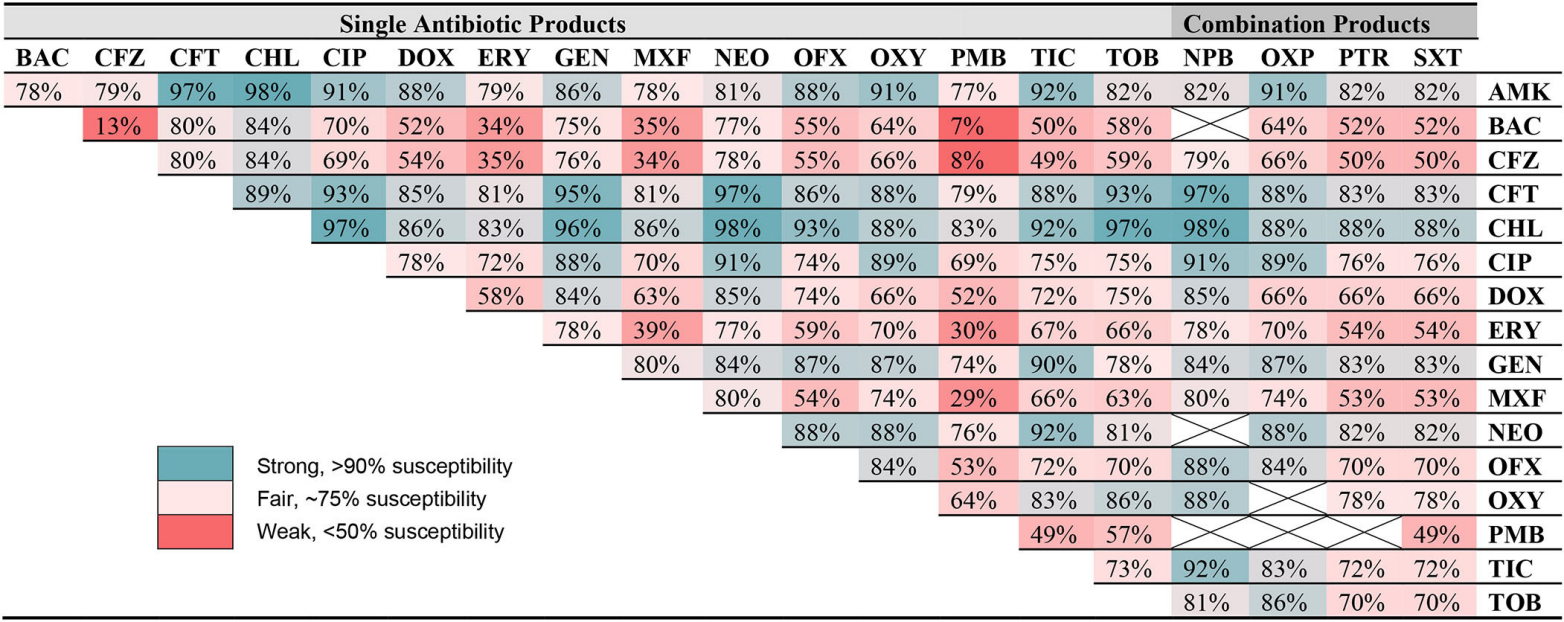

a *Antibiotics used: AMK, amikacin; BAC, bacitracin; CFZ, cefazolin; CFT, ceftiofur; CHL, chloramphenicol; CIP, ciprofloxacin; DOX, doxycycline; ERY, erythromycin; GEN, gentamicin; MXF, moxifloxacin; NEO, neomycin; OFX, ofloxacin; OXY, oxytetracycline; PMB, polymyxin B; TIC, ticarcillin; TOB, tobramycin; NPB, neomycin, polymyxin B, bacitracin; OXP, oxytetracycline, polymyxin B; PTR, polymyxin B, trimethoprim; SXT, sulfamethoxazole, trimethoprim; crossed out boxes indicate combinations where the same antibiotic would be used twice*.

### Multi-Drug Resistant Isolate Characteristics

Bacteria considered MDR represented 20% (69/352) of all canine isolates. Specifically, MDR strains were identified in 33% (40/123) of *Staphylococcus* sp., 25% (17/68) of *Streptococcus* sp., 19% (5/27) of *Corynebacterium* sp., 10% (1/10) of *E. coli*, 0% (0/44) of *Pseudomonas* sp., 0% (0/17) of *Pasteurella* sp., and 10% (6/63) of all other isolates. Notably, the incidence of MDR increased over the last 5 years of data collection, with MDR strains representing 5% (2/42) of all bacteria isolated in 2016, 10% (13/126) in 2017, 22% (12/54) in 2018, 32% (17/53) in 2019, and 34% (11/32) in 2020. Dogs with a high prevalence of MDR isolates included shih tzus (13/46, 28%), mixed breed (10/23, 43%), pugs (3/9, 33%), and Boston terriers (2/16, 13%). Compared to mixed breed dogs, Pomeranians and Saint Bernards were significantly more likely to yield a MDR isolate when bacterial growth was present, with 3/3 isolates classified as MDR for Pomeranians (OR = ∞, *P* = 0.049) and 4/4 isolates classified as MDR for Saint Bernards (OR = ∞, *P* = 0.019).

## Discussion

In the present study, the three most common bacterial genera isolated from dog corneas were *Staphylococcus* (32.3%), *Streptococcus* (19.1%), and *Pseudomonas* (12.5%). These findings are generally in agreement with other canine studies across the globe (2, 3, 9–11), with subtle geographic differences such as a lower prevalence of *Staphylococcus* species in Australia (8) or a relatively higher prevalence of *Streptococcus* species in the Midwestern United States (present study) compared to other locations (6, 7). *Staphylococcus pseudintermedius* was the most common *Staphylococcus* species (26.7% of all isolates), as recognized in most canine reports (2, 3, 7, 10, 11), followed by non-specified Coagulase-negative *Staphylococcus* species (3%) and *Staphylococcus epidermidis* (0.6%). Surprisingly, *Staphylococcus aureus* only accounted for 0.4% of isolates in the present study, while it had previously been reported as more prevalent in canine bacterial keratitis (6, 9). *Streptococcus canis* (12%) and *Pseudomonas aeruginosa* (7.5%) were the most common species cultured for each respective genus, consistent with previous reports (3, 8, 10, 11).

As compared to mixed-breed dogs, one canine breed (Labrador retriever) was found to be less likely to present to the Ophthalmology service with bacterial keratitis—a finding presumably related to the higher proportion of eye certification exams in Labradors—while eight canine breeds were found to be at higher risk. Over-represented dogs included brachycephalic breeds (Boston terrier, Cavalier King Charles spaniel, pug, and shih tzu), as previously reported (2, 8, 9), but also non-brachycephalic breeds such as Saint Bernard, miniature pinscher, rat terrier, and silky terrier. Further, the study assessed the seasonality of bacterial isolates and found that summer increased the risk of yielding a positive bacterial culture from canine corneas, notably *Pseudomonas* species. Summer was also shown to affect the prevalence of selected bacterial isolates in humans (22). UV light is a known risk factor for potentiating infections in bovine eyes (23), resulting in degenerative changes to corneal epithelial cells that allow for easier bacterial colonization (24, 25), and the same may be true in dogs. Awareness of seasonal variation might inform clinical recommendations and prevention strategies, and it is therefore advised to report seasonality in bacteriological studies.

Appropriate management of corneal ulcers requires an understanding of common bacterial isolates and associated susceptibilities to antibiotics. Monotherapy (i.e., individual drug or combination drug such as triple antibiotic) is generally advised for ulcers characterized as “simple” (superficial, no gross signs of infection), while combination therapy with 2 antibiotic formulations is generally suggested for “complicated” corneal ulcers with active signs of infection (e.g., stromal loss, keratomalacia, cellular infiltrates) to provide broad spectrum coverage for both gram-positive and gram-negative bacteria (12–15). Further, the complementary use of a systemic antibiotic can be considered in well-vascularized corneal lesions and corneal perforations (at risk for endophthalmitis), or when systemic administration achieves tear film concentrations above minimal inhibitory concentrations for common bacterial isolates. The latter was demonstrated for various antibiotics administered parenterally to cows (i.e., oxytetracycline, chloramphenicol, gentamicin, and erythromycin) (26) but has not been documented in companion animals to date. The summary information detailed in the manuscript's tables provide a quick reference for clinicians that manage bacterial keratitis in canine patients.

Individual antibiotics with the highest efficacy rates included chloramphenicol (83%), ceftiofur (79%), amikacin (77%), neomycin (76%), and gentamicin (74%). When considering commercially available formulations, the best option remains triple antibiotic (neomycin-polymxin B-bacitracin) with 77% efficacy, consistent with general guidelines to use this medication for prophylactic use in non-infected ulcers (4, 5, 13, 27), followed by gentamicin (4, 13). Other options for empirical monotherapy include chloramphenicol, ceftiofur, and amikacin (≥77% efficacy), however chloramphenicol and amikacin require compounding in the US, while ceftiofur is only marketed for horses and production animals as an injectable formulation for parenteral use, with proven clinical efficacy for cattle with infectious bovine keratoconjunctivitis (28). Chloramphenicol and ceftiofur were highly effective against *Streptococcus* and *Staphylococcus* species (≥88%), but their efficacy against *Pseudomonas* was limited (20%). Preferred antibiotics for *Pseudomonas* species included ciprofloxacin (95%) and aminoglycosides (amikacin, gentamicin, neomycin) with ≥93% efficacy. It is important to note that another 2nd-generation fluoroquinolone (ofloxacin) had relatively limited efficacy against *Pseudomonas* species in the present study (45%), consistent with the trend described in some veterinary and human studies (29, 30). As expected, the MIC_50_ and MIC_90_ values for *Pseudomonas* sp. and cephalosporins (cefazolin and ceftiofur) were higher than MIC values detected for *Staphylococcus* and *Streptococcus* species; further, MIC_50_ and MIC_90_ values for *Streptococcus* sp. and aminoglycosides (amikacin, gentamicin, neomycin, and tobramycin) were higher than MIC values identified for *Staphylococcus* or *Pseudomonas* isolates, as recently described in canine and equine patients (31).

Combination antibiotic therapies are routinely used in practice to provide broad-spectrum coverage for suspected infected corneal ulcers (12–15), yet the efficacy of combined therapy is not addressed in previous bacteriological studies. A strong combination therapy highlighted in the present study is chloramphenicol (*Staphylococcus, Streptococcus, Corynebacterium*, and “others” coverage) and ciprofloxacin (*Pseudomonas* coverage), two drugs with good penetration into corneal tissues (32) and excellent efficacy against common bacterial isolates in dogs when used together (97%). Other strong combinations include chloramphenicol-amikacin (98%), chloramphenicol-tobramycin (97%), amikacin-ceftiofur (97%), chloramphenicol-gentamicin (96%), and ciprofloxacin-gentamicin (96%). Importantly, adjustments to combination therapies should be considered when culture and sensitivity results are obtained, avoiding the unnecessary use of antibiotics, and reducing ocular surface toxicity from excessive use of preservative-containing ophthalmic solutions.

Systemic antibiotics can be used to complement topical antibiotic therapy in dogs with bacterial keratitis if the lesion is vascularized or the cornea is perforated. Amoxicillin-clavulanic acid was very effective for gram-positive organisms (≥83% efficacy) (16), while aminoglycosides and other selected antibiotics (ceftazidime, marbofloxacin, and piperacillin) were most effective against gram-negative organisms (≥76% efficacy). Antibiotics generally considered “last-line” therapies such as vancomycin (90%) and imipenem (86%) were highly effective against most bacterial isolates.

Antimicrobial resistance is a serious concern in veterinary ophthalmology (33). The overall prevalence of MDR isolates was relatively high (20%) in the present study, with Pomeranians and Saint Bernards being significantly more likely to yield MDR isolates when presented to our Ophthalmology service with corneal ulcers. The high prevalence of MDR isolates in these two canine breeds is puzzling and may simply be related to low sample size; alternatively, it is possible the microbiome differs among canine breeds due to peculiarities in ocular surface anatomy and physiology. Interestingly, none (0%) of *Pseudomonas* isolates were considered MDR in this study, likely due to extensive intrinsic resistance profile for this bacterial genera in veterinary medicine (18, 21); of note, most (>90%) *Pseudomonas* isolates were susceptible to aminoglycosides and ciprofloxacin as previously described (7, 34). Further, we noted an alarming, escalating trend of multi-drug resistance over the last 5 years of the study (5% in 2016 to 34% in 2020). MDR is also prevalent in human patients with bacterial keratitis (35, 36). The “Antibiotic Resistance Monitoring in Ocular Microorganisms” (ARMOR) studies in humans have provided invaluable information for clinicians managing bacterial keratitis in practice, and the same collaborative effort is critically needed in veterinary medicine to mitigate the rise of MDR in animals. Such work could promote antimicrobial stewardship at a regional- or clinic-level, decreasing the rate of MDR through judicious use of ophthalmic antibiotics in practice.

The present work focused on aerobic bacterial cultures in dogs with ulcerative keratitis. Depending on the clinical appearance of the lesion, clinicians should also consider anaerobic and/or fungal cultures to increase the likelihood of identifying the causative agent (37, 38). In fact, diagnostic tests other than culture-based methods will soon become the gold standard for microbial species identification as the field of clinical microbiology is rapidly evolving. Unlike culture-based methods, technologies such as mass spectrometry and nucleic acid sequencing provide rapid and sensitive tools to probe the microbiome in clinical patients (39)—as recently described for the ocular surface of veterinary species (40, 41)—enabling clinicians to optimize the antibiotic treatment sooner and thereby improve clinical outcomes (42).

Similar to previous reports of antibiotic susceptibility profiles in veterinary medicine, the main limitation of the present study is the reliance on incomplete veterinary specific CLSI guidelines to determine whether a bacterial isolate is susceptible or resistant to a given antibiotic. In veterinary CLSI guidelines, the lack of interpretative breakpoints for selected bacteria/antibiotic combinations yields a “non-interpretable” clinical interpretation in the antibiogram. Such missing information may be due to intrinsic resistances, or the absence of pharmacokinetics-pharmacodynamics studies that assess the concentration of a given antibiotic at the target tissue and determine whether such levels are above the minimal inhibitory concentration (MIC) of a given bacterial species. Findings presented herein likely underestimate the true susceptibility rates as some of the “non-interpretable” results may in fact be sensitive in clinical practice. For instance, moxifloxacin susceptibility rates were generally low in the present study because 64% of moxifloxacin results were reported as “non-interpretable” by the CLSI guidelines, while in practice this fourth-generation fluoroquinolone is considered superior to ofloxacin and ciprofloxacin for most bacterial isolates (43). Similarly, polymyxin B, cefazolin, and bacitracin also had high numbers of “non-interpretable” reports (72, 71, and 55%, respectively). On the other hand, caution must be exercised when clinical guidelines provide an actual interpretation of “susceptible” or “resistant.” A closer look at the MIC data provided by the ophthalmic panel (JOEYE2 plate) shows that the concentration tested for selected antibiotics (e.g., 4 μg/mL for chloramphenicol/neomycin/tobramycin, 16 μg/mL for amikacin/ticarcillin) is relatively high when compared to drug levels achieved in canine tear film. Indeed, topical drug delivery achieves high concentrations in the short-term but concentrations on the canine ocular surface rapidly decrease due to efficient drainage through the nasolacrimal duct (44–46).

In conclusion, the bacterial profile from corneal cultures in Iowa and surrounding Midwestern United States followed world-wide trends with high proportion of *Staphylococcus, Streptococcus*, and *Pseudomonas* species. Characteristics of bacterial keratitis in dogs were influenced by season and canine breeds. The rate of multi-drug resistance was relatively high, notably for *Staphylococcus* isolates, with an alarming escalating trend over time. Appropriate selection of empiric antibiotic therapy is important to enhance therapeutic outcome and reduce antibacterial resistance in dogs with corneal ulceration, whether using individual or combination drug therapy. Subsequently, clinicians' selection of antibiotics should be guided by the antibiogram received for each given patient, requiring adjustments to the empirical therapy initiated earlier in selected cases.

## Data Availability Statement

The raw data supporting the conclusions of this article will be made available by the authors, without undue reservation.

## Ethics Statement

Ethical review and approval was not required for the animal study because the work was a retrospective analysis of bacterial cultures collected in dogs as part of the patients' standard of care. Written informed consent for participation was not obtained from the owners because the work was a retrospective analysis of bacterial cultures collected in dogs as part of the patients' standard of care.

## Author Contributions

LS and RA conceptualized and designed the study in consultation with JH and DK. LS and JH analyzed the data. All authors wrote the manuscript.

## Conflict of Interest

The authors declare that the research was conducted in the absence of any commercial or financial relationships that could be construed as a potential conflict of interest.
